# Cutting through complexity: the Breathing, Thinking, Functioning clinical model is an educational tool that facilitates chronic breathlessness management

**DOI:** 10.1038/s41533-021-00237-9

**Published:** 2021-05-10

**Authors:** Anna Spathis, Julie Burkin, Catherine Moffat, Rachel Tuffnell, Stephen Barclay, Jonathan Mant, Sara Booth

**Affiliations:** 1 Cambridge University Hospitals NHS Foundation Trust, Cambridge, UK; 2 University of Cambridge, Cambridge, UK

**Keywords:** Respiratory signs and symptoms, Quality of life, Palliative care

## Abstract

Chronic breathlessness is a distressing symptom that is challenging to manage. The Breathing, Thinking, Functioning clinical model is an educational tool developed to support breathlessness management. Health professionals report that the model increases clinician and patient understanding of this complex symptom, and provides a simple and structured approach to personalised self-management.

Breathlessness is a debilitating symptom experienced by over a quarter of community-dwelling older adults, and most people with chronic respiratory conditions^1,2^. The global prevalence is rising with ageing populations and increasing multi-morbidity^3^. COVID-19 is further exacerbating the problem, with evidence that a significant minority experience persistent breathlessness long after the acute infection has resolved^4^.

Primary care is recognised to be particularly well placed to support chronic breathlessness management^5,6^. However, health professionals in both primary care and respiratory medicine describe feeling ‘out of their depth’ and ‘overwhelmed’, when attempting to control a symptom that persists despite optimal disease management^5,7^. Breathlessness is, indeed, challenging to manage. Its severity bears little relationship to that of the underlying condition, with more than 40% of patients with mild COPD, for example, experiencing moderate to severe breathlessness^8^. Drug treatments tend not to help. Opioids are often prescribed, but were found to be ineffective in a recent definitive trial^9^, and are associated with adverse effects and increased mortality^10–12^. There is no evidence benzodiazepines improve breathlessness^13^.

Non-drug measures are more effective but under-used^14^. Evidence-based approaches, such as exercise, cognitive behavioural therapy, activity management, mindfulness and facial cooling, are hindered by limited patient engagement in self-management and lack of professional expertise^15–19^. Pulmonary rehabilitation is frequently inaccessible, particularly to those with the worst symptoms, with only two out of five people referred completing a course^20^. Sporadic specialist breathlessness management programmes have emerged over the last decade. Although effective, these have very little capacity and are mostly accessed by patients with advanced cancer^21^.

Improving access to breathlessness management support is vital. Recent consensus exercises have led to increasing calls to prioritise the provision of education to upskill generalist health professionals and ‘…embed core therapeutic components into routine clinical practice’^22,23^. Despite this, there has been a conspicuous lack of breathlessness management education developed for health professionals, with the only two previous publications describing multi-day experiential clinical skills workshops^24,25^.

The Cambridge Breathlessness Intervention Service has developed the only educational tool to support chronic breathlessness management^26^. The Breathing, Thinking, Functioning (BTF) clinical model (Fig. 1) conceptualises emotional and behavioural responses to breathlessness, leading to vicious cycles that inadvertently worsen the symptom or perpetuate it beyond a trigger. It explains the disassociation between the severity of breathlessness and the underlying condition, and provides a rationale for symptom management: unlike drug treatments, non-drug approaches can break a vicious cycle, turning it into a cycle of health improvement. Each part of the model is evidence-based; for example, it is well-established that misinterpretations of bodily sensations can lead to a spiral of deteriorating symptoms and panic^27^.

**Fig. 1:**
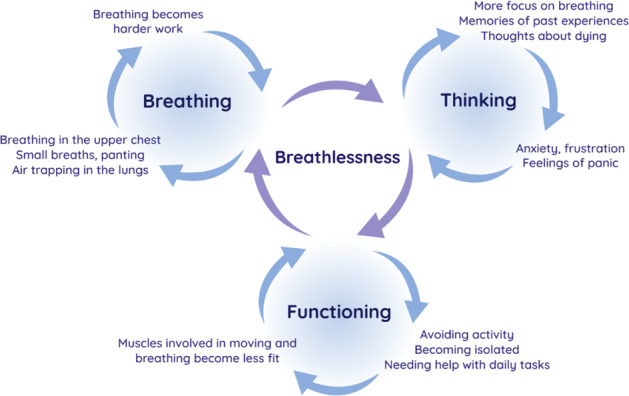
Breathing, Thinking, Functioning clinical model. The model conceptualises vicious cycles of instinctive emotional and behavioural responses to breathlessness.

International interest in the BTF model suggests high face validity and a number of centres have incorporated the model into their teaching^28^. This communication describes feedback from health professionals who have received breathlessness management training based on the model.

Of 209 delegates, 179 completed the pre-course questionnaire. The three-month post-course questionnaire was sent to the 164 participants providing valid email addresses, and 109 responded. Most participants were allied health professionals (37%) or nurses (36%), with the remainder being doctors (10%) or of unknown discipline (16%). Of the 85% with a known working context, more than half (57%) worked in respiratory or primary care.

Participants were significantly more confident in managing breathlessness three months after attending the study day compared to baseline (73% vs. 25% rated confidence as ‘quite a bit’ or ‘very much’; Mann–Whitney *U* test, *p* < 0.001). They also felt more experienced in managing breathlessness (70% vs. 37% rated the experience as ‘quite a bit’ or ‘very much’; *p* < 0.001) and were using the BTF model more frequently (57% vs. 10% rated level of use as ‘quite a bit’ or ‘very much’; *p* < 0.001). Most participants considered the BTF model to be valuable in their clinical practice, rating its usefulness as ‘very much’ (45%) or ‘quite a bit’ (36%), and only a few ‘a little bit’ (3%) or ‘not at all’ (1%).

The majority of the free-text responses (72/118) described useful aspects of the model. The most consistent theme (36 comments) related to the model making sense of breathlessness in terms of its drivers and management. The emphasis was on patients’ understanding, with a number also alluding to professional understanding. The model was repeatedly described as being easy to understand and use, with it making ‘sense to staff and patients’. Patients’ increased understanding and insights, in turn, appeared to help them self-manage breathlessness non-pharmacologically, allowing them to ‘try to change things and take back control’.

The second strongest topic of feedback, reflected in 16 comments, was the model’s practical value in structuring and focusing breathlessness management. The ‘logical’ structured approach was helpful both during symptom assessment and management. It helped to ‘divide breathlessness up’ so it felt more feasible to manage. The inherent structure, in turn, was considered by many to promote individualised care, focused on the specific needs of each person. Participants commented on the ‘flexibility of the approach’ so they could ‘tailor to the individual’, helping them ‘identify the key problem areas and know where to start’. Several participants alluded to it being ‘holistic’, particularly valuing the ‘emphasis on emotional components’.

In relation to amendments, the most common response was that no changes were needed to the model (*n* = 30). The remaining comments (*n* = 19) fell into two themes, the main one being participants’ requests for BTF model resources, with ‘flash cards’ or ‘pocket sized’ cards that could be used with, and given to, patients; others suggested the development of an ‘App’ based on the model. Five participants commented on the lack of time in short patient consultations and the need for brief intervention, particularly in a primary care context.

The BTF clinical model was perceived to be a valuable educational tool for health professionals managing chronic breathlessness. Three months after attending one study day, most participants felt more confident in their ability to manage the symptom and were finding the model useful in their clinical practice. Qualitative data provided further insights: the role of the model in making sense of the symptom, and its capacity to provide an easy, structured and personalised approach to breathlessness management. Overall, the outcomes suggest that the fundamental benefit of the model for health professionals comes from incising through the complexity of this challenging symptom.

These findings are of particular pertinence to primary care, which holds much of the burden of long-term conditions. Patients experience symptoms, not diseases. With incurable disease and escalating multimorbidity, symptom-focused rather than disease-focused approaches become increasingly relevant. The BTF model supports breathlessness self-management by facilitating workforce training that allows staff and patients to make sense of the symptom and enables structured, personalised intervention.

The request for App development has the potential to mitigate the concern about time-limited patient consultations. Healthcare digital transformation is accelerating, and electronic resources support self-management and increase health service efficiency. The development of a BTF App is in progress. The finding that the model supports individually focused management reinforces its potential role in brief intervention.

Chronic breathlessness is a global phenomenon. Breathlessness management training and digital tools will not only improve access to symptom control in high-income countries but can also support the type of low-intensity, low-resource interventions urgently needed in less wealthy nations. The COVID-pandemic is rapidly intensifying the problem, changing the need for access to effective breathlessness intervention from important to imperative.

## Methods

The Cambridge Breathlessness Intervention Service delivers single study days, underpinned by the BTF model, for any health professional wishing to learn about chronic breathlessness management. Lectures in the morning explain the BTF model and management of each of the three vicious cycles; in the afternoon, participants rotated through three experiential workshops. Participants at four of the study days (2017-19) completed a brief questionnaire at the start of the day self-rating experience and confidence in breathlessness management, and any previous use of the model, using three 5-item verbal rating scales (not at all, a little bit, somewhat, quite a bit, very much). Provision of an email address gave implicit consent to be sent a follow-up questionnaire three months after the study day. At this point, participants completed the same questionnaire with an additional question about the perceived usefulness of the model, along with free text feedback on useful aspects of, and suggested changes to, the model. Research ethics approval was not required as this was an education evaluation that set out to gain feedback to improve education, rather than derive generalisable knowledge, and it did not involve students or patients^29^.

### Reporting summary

Further information on research design is available in the Nature Research Reporting Summary linked to this article.

## Supplementary information

Reporting Summary

## Data Availability

All relevant data are available from the authors.

## References

[1] Currow DC, Clark K, Mitchell GK, Johnson MJ, Abernethy AP (2013). Prospectively collected characteristics of adult patients, their consultations and outcomes as they report breathlessness when presenting to general practice in Australia. PLoS ONE.

[2] Currow DC (2017). Chronic breathlessness associated with poorer physical and mental health-related quality of life (SF-12) across all adult age groups. Thorax.

[3] Etkind S (2017). How many people will need palliative care in 2040? Past trends, future projections and implications for services. BMC Med..

[4] Greenhalgh T, Knight M, A’Court C, Buxton M, Husain L (2020). Management of post-acute covid-19 in primary care. BMJ.

[5] Baxter N (2017). Breathlessness in the primary care setting. Curr. Opin. Supp. Palliat. Care.

[6] Elliott-Button HL, Johnson MJ, Nwulu U, Clark J (2020). Identification and assessment of breathlessness in clinical practice: a systematic review and narrative synthesis. J. Pain. Symptom Manag..

[7] Lunn S, Dharmagunawardena R, Lander M, Sweeney J (2019). It’s hard to talk about breathlessness: a unique insight from respiratory trainees. Clin. Med..

[8] Mullerova H, Chao L, Hao L, Tabberer M (2014). Prevalence and burden of breathlessness in patients with chronic obstructive pulmonary disease managed in primary care. PLoS ONE.

[9] Currow D (2020). Regular, sustained-release morphine for chronic breathlessness: a multicentre, double-blind, randomised, placebo-controlled trial. Thorax.

[10] Barnes P, McDonald J, Smallwood N, Manser R (2016). Opioids for the palliation of refractory breathlessness in adults with advanced disease and terminal illness. Cochrane Database Syst. Rev..

[11] Vozoris NT (2016). Incident opioid drug use among older adults with chronic obstructive pulmonary disease: a population-based cohort study. Br. J. Clin. Pharmacol..

[12] Ekström MP, Bornefalk-Hermansson A, Abernethy AP, Currow DC (2014). Safety of benzodiazepines and opioids in very severe respiratory disease: national prospective study. BMJ.

[13] Simon ST (2016). Benzodiazepines for the relief of breathlessness in advanced malignant and non-malignant diseases in adults. Cochrane Database Syst. Rev..

[14] Smallwood N (2018). Differing approaches to managing the chronic breathlessness syndrome in advanced COPD: a multi-national survey of specialists. COPD.

[15] Livermore N (2015). Cognitive behaviour therapy reduces dyspnoea ratings in patients with chronic obstructive pulmonary disease (COPD). Respir. Physiol. Neurobiol..

[16] Kako J (2018). Fan therapy is effective in relieving dyspnea in patients with terminally ill cancer: a parallel-arm, randomized controlled trial. J. Pain. Symptom Manag..

[17] Tan S (2019). The effect of 20-minute mindful breathing on the rapid reduction of dyspnea at rest in patients with lung diseases: a randomized controlled trial. J. Pain. Symptom Manag..

[18] Galbraith S, Fagan P, Perkins P, Lynch A, Booth S (2010). Does the use of a handheld fan improve chronic dyspnea? A randomized, controlled, crossover trial. J. Pain. Symptom Manag..

[19] Malpass A, Feder G, Dodd JW (2018). Understanding changes in dyspnoea perception in obstructive lung disease after mindfulness training. BMJ Open Respiratory Res..

[20] Steiner, M. et al. Pulmonary Rehabilitation: steps to breathe better. National Chronic Obstructive Pulmonary Disease (COPD) Audit Programme: Clinical audit of Pulmonary Rehabilitation services in England and Wales 2015. National clinical audit report. (Royal College of Physicians, London. 2016).

[21] Brighton LJ (2019). Holistic services for people with advanced disease and chronic breathlessness: a systematic review and meta-analysis. Thorax.

[22] Brighton LJ (2019). Recommendations for services for people living with chronic breathlessness in advanced disease: results of a transparent expert consultation. Chron. Respir. Dis..

[23] Williams MT (2019). Chronic breathlessness explanations and research priorities: findings from an international delphi survey. J. Pain. Symptom Manag..

[24] Shaw V, Davies A, Ong BN (2019). A collaborative approach to facilitate professionals to support the breathless patient. BMJ Supp. Pall. Care.

[25] Froggatt K, Walford C (2005). Developing advanced clinical skills in the management of breathlessness: evaluation of an educational intervention. Eur. J. Oncol. Nurs..

[26] Spathis A (2017). The Breathing, Thinking, Functioning clinical model: a proposal to facilitate evidence-based breathlessness management in chronic respiratory disease. NPJ Prim. Care. Respir. Med..

[27] Teachman B (2010). Catastrophic misinterpretations as a predictor of symptom change during treatment for panic disorder. J. Consult Clin. Psychol..

[28] Johnston K (2020). Attitude change and increased confidence with management of chronic breathlessness following a health professional training workshop: a survey evaluation. BMC Med. Educ..

[29] Health Research Authority. *UK Policy Framework For Health And Social Care Research*. https://www.hra.nhs.uk/planning-and-improving-research/policies-standards-legislation/uk-policy-framework-health-social-care-research/uk-policy-framework-health-and-social-care-research/ (2021).

